# The diagnostic quality of needle brain biopsy specimens obtained with different sampling methods – Experimental study

**DOI:** 10.1038/s41598-019-44622-4

**Published:** 2019-05-30

**Authors:** Piotr Trojanowski, Bożena Jarosz, Dariusz Szczepanek

**Affiliations:** 1 Department of Otolaryngology and Laryngological Oncology, Medical University of Lublin, Jaczewskiego 8, 20 954 Lublin, Poland; 2 Department of Neurosurgery and Paediatric Neurosurgery, Medical University of Lublin, Jaczewskiego 8, 20 954 Lublin, Poland

**Keywords:** Surgical oncology, CNS cancer

## Abstract

The aim is to examine whether brain tissue samples obtained through needle biopsy are better for histopathological evaluation when obtained with defined vacuum pressure, a novel needle rotation method, and using different needle type - Laitinen or Nashold. Moreover the paper aims to answer the question: Does vacuum and mechanical injury resulting from different sampling methods damage the tissue specimen challenging the diagnosis?. Eight hundred biopsy specimens from fresh swine brains were obtained using Nashold and Laitinen brain biopsy needles through inner cannula cutting or needle rotation sampling at vacuum pressure, from 0 to 0.06 MPa. The specimen weight and tissue quality for microscopic assessment were evaluated using the Mair score. Rising aspiration pressure increased the biopsy sample weight. Needle rotation delivered larger biopsy samples than the standard method. Laitinen provided larger samples than the Nashold needle, with the same sampling method or vacuum pressure. A higher histopathological diagnostic quality of tissue was obtained with the Laitinen needle than with Nashold, with higher vacuum pressure than lower pressure and finally with needle rotation than the standard method. No tissue damage caused by higher suction pressure or method of tissue separation was documented. Brain tissue samples obtained through needle biopsy are better for histopathological evaluation when obtained with higher vacuum pressure, a novel needle rotation method and with Laitinen needle. Higher suction pressure and sampling methods did not cause tissue damage.

## Introduction

Stereotactic needle biopsy plays an important role in establishing histological diagnosis, which is essential in the proper management of many brain diseases. Its diagnostic yield is high, usually above 80%. The diagnostic accuracy ranges from 73% to 97%; the differences may result from the lack of standardized criteria of diagnostic yield^1–8^. Non-diagnostic samples in brain tumour biopsies are reported in up to 24% of cases and diagnostic errors in 10–30% of cases^2–4,9^. Various techniques are employed to improve the diagnostic value of brain biopsy methods^10–18^.

Targeting accuracy in brain biopsies is an important concern, but tissue sample quality for the pathological examination is equally significant. Modern imaging based on magnetic resonance fused with proton emission tomography sometimes provides good coordinates for high accuracy stereotactic and robot-assisted aiming devices, which substantially reduce the risk of missing pathological areas^14,16,17,19^.

The safe acquisition of an adequate quantity of undamaged tissue with a biopsy needle remains an area with much scope for improvement. Modifications in biopsy needle construction and sampling technique may serve this purpose and offer a space for investigations.

Needle biopsy, utilised for various parenchymal organs, often uses suction to obtain tissue samples^20–24^. In the context of brain biopsy, it has not been studied. However, some investigators mention slight vacuum pressure being applied in a brain biopsy with a syringe to pull tissue into the needle^25–27^. In the majority of publications, the level of vacuum pressure is neglected and its subsequent effect on sample volume and quality due to possible mechanical damage has not been analysed. There are only a few papers that address this subject of needle biopsy in parenchymal structures^21,24,28–30^.

In existing literature, the influence of the execution of needle brain biopsy has not been specifically examined with respect to its impact on the diagnostic quality of the resulting histopathological preparations. Therefore, the present study has been designed to evaluate the effectiveness of brain needle biopsy in obtaining tissue samples and in the assessment of their histopathological quality in relation to the type of needle, method of tissue sampling and the applied level of vacuum.

## Material and Methods

The experiments were carried out on 100 fresh whole swine brains (Sus scrofa f. domestica), obtained immediately after commercial slaughtering in an abattoir (Lubmeat, Lublin). The commercial slaughtering house Lubmeat operates under the state regulations according to EU guidelines. The University Ethical Committee considered this research not needing specific ethical approval, since the tissue has been obtained in the routine abattoir procedure without any additional intervention.The brains were transferred into saline and the biopsies were performed within 60 minutes.

**Figure 1:**
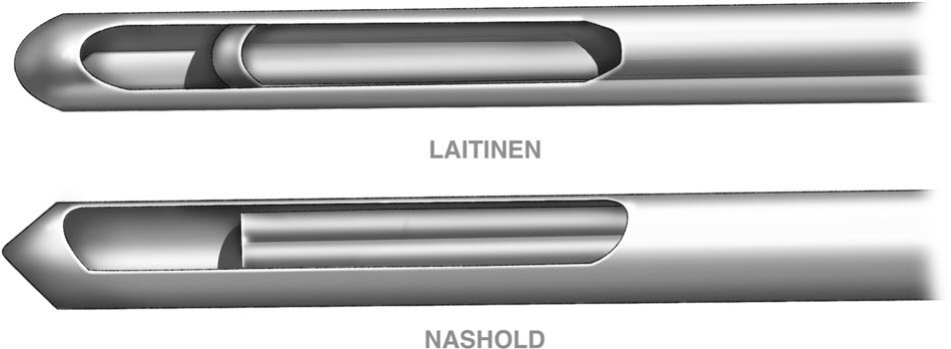
Different shapes of the window and inner cannula in the Laitinen and Nashold biopsy needles. The pressure gradient between the tissue and the needle lumen, promoting tissue displacement into the needle, was increased through aspiration with a 60 ml silicone piston syringe, Monoject (UK), connected to the needle via a 3 mm tube (Pressure line, Viggo-Spectramed). The drain withstands vacuum of 90 KPa. (A) three-way cock allowed the connection of a pressure gauge (Mera KFM) scaled with 0.002 MPa accuracy from 0–0.1 MPa. The syringe piston was moved with a precise lever to set one of the four vacuum pressures of 0.01, 0.02, 0.04, 0.06 MPa. The biopsy needle with the closed window was inserted through a fixed tube of a stereotactic biopsy holder to a depth of 2.5 cm perpendicular to the brain surface. The three-way stopcock was positioned to connect the needle to the negative pressure line. The window was opened, and the tissue sample was cut employing the standard or rotation methods. After sampling, the window was closed with the inner cannula and the pressure returned to normal. The tissue samples were flushed out of the needle with saline into a vial containing formaldehyde (4% aqueous solution of formic aldehyde and 1% calcium carbonate).

Two types of brain biopsy needles for stereotactic biopsies were used. The Sedan type of needle, Laitinen (Umea, Sweden) has an external diameter of 2.0 mm, lumen diameter of 1.5 mm and total length of 25.4 cm. The needle tip is blunt. A rectangular window 5.5 × 1.5 mm is located on the side of the needle, 1.5 mm from the tip. The window can be closed using a thin-walled, inner cannula, sliding inside the needle lumen (Fig. 1). The sharp edges of the inner cannula tip act in the manner of a guillotine, cutting the tissue bulging into the window. The degree of bulging and, thereby, the sample volume depends on the physical properties of the tissue as well as on the pressure gradient between the brain and the needle lumen. The negative vacuum pressure applied to the needle promotes the bulging of the tissue into the needle lumen.

The second needle, the commonly used Nashold biopsy needle, Radionics (USA), has a smaller sampling window of 9.5 × 1.2 mm, located 2 mm from the needle tip. The needle diameter is 1.8 mm, lumen is 1.5 mm and the total length is 29.8 cm. The tip of the needle is dome shaped. The inner cannula has a window, which can be aligned with the outer cannula window through rotation. In this position, the tissue bulges into the needle lumen. Further rotation of the inner cannula cuts off the tissue displaced into the needle and closes the window. The removal of the inner cannula from the needle delivers a piece of tissue for examination. This sampling is referred to as the standard method.

Both needle types were also used in a novel method called needle rotation. The needle with a closed window was advanced into the brain, the window was opened and the needle was rotated by 360 degrees. Tissue separation was achieved through the cutting by the longer window edge. The window was closed and the needle removed.

Both needles share a similar shape. The sampling window dimensions and the sample cutting mechanisms are different (Fig. 1).

Each of the 100 brains was subjected to 4 biopsies from each brain hemisphere at the frontal, parietal, temporal and occipital region. This delivered 50 samples each, obtained with the same combination of parameters: vacuum pressure, sampling method and needle type. A single investigator performed all 800 biopsies. The samples were fixed in 10% neutral buffered formalin, and after 24 hours, they were weighed with an accuracy of 0.01 g.

Every tenth sample was processed to obtain a paraffin block, which were then used to prepare haematoxylin and eosin stained slides. The slides were examined under a light microscope by one pathologist to assess their quality for histopathological diagnosis, based on the Mair scale (Table 1). The Mair scale identifies cellular contents of the sample, the degree of damage to the cell structure, the level of tissue architecture preservation and contamination with blood clots^31^. The diagnostic utilities of the samples obtained with different biopsy methods were compared considering the sum of scored points. A cumulative score for each specimen was classified into one of the following three categories: score 0–2: unsuitable for diagnosis; score 3–6: adequate for cytological diagnosis; score 7–10: diagnostically superior^31^.

**Table 1:** The scoring system for evaluation of diagnostic quality of biopsy tissue samples for light microscopy following Mair *et al*.^31^.

	Criterion	Qualitative description	Score
1.	Background blood/clot	Large amount/great compromise to diagnosis	0
		Moderate/diagnosis possible	1
		Minimal/diagnosis easy; specimen of textbook quality	2
2.	Amount of cellular material	Minimal to absent/diagnosis not possible	0
		Sufficient for diagnosis	1
		Abundant/diagnosis simple	2
3.	Degree of cellular degeneration	Marked/diagnosis impossible	0
		Moderate/diagnosis possible	1
		Minimal/good preservation; diagnosis easy	2
4.	Degree of cellular trauma	Marked; diagnosis impossible	0
		Moderate; diagnosis possible	1
		Minimal; diagnosis easy	2
5.	Retention of appropriate architecture	Minimal to absent/non diagnostic	0
		Moderate/some preservation	1
		Excellent architecture display, closely reflecting histology	2
6.	Total		10

The mean weight of the 50 specimens obtained with the same combination of parameters of vacuum pressure, sampling method and needle type was evaluated statistically using the Student’s t-test for independent samples. The difference was regarded statistically significant when p < 0.001.

Some biopsies did not deliver enough tissue to enable the preparation of a histopathological slide. In the existing literature, these are classified as zero-biopsies^32^. One of the measures of the biopsy’s effectiveness in this regard is the number of biopsy attempts necessary to obtain a sample suitable for histological examination. In order to measure this aspect of effectiveness, the number of attempts to obtain 50 valuable samples was noted.

## Results

The experiments performed provided data to generate the following results. The mean sample weight obtained with the Laitinen and Nashold biopsy needles, varying based on standard or rotation method of sampling and levels of vacuum pressure, is presented in Table 2. The samples acquired with the same combination of biopsy method and vacuum pressure were larger with the Laitinen needle than with the Nashold one. In a majority of cases, this difference was statistically significant. The mean weight of samples obtained with Laitinen was 26.32 mg, whereas that with the Nashold needle was 13.85 mg.

**Table 2:** Mean weight of a sample obtained with the Laitinen or Nashold needles in relation to standard or rotation method and various levels of vacuum pressure. Each mean value was calculated from 50 samples.

Sampling method	Vacuum pressure level MPa	Needle				p < 0.01
		Laitinen		Nashold		
		Weight (mg)	SD	Weight (mg)	SD	
Standard	0.00	**7.18**	3.89	**3.93**	1.57	*
	0.02	**10.45**	4.2	**9.51**	3.42	
	0.04	**20.63**	6.94	**14.26**	6.54	*
	0.06	**28.77**	9.43	**15.77**	7.45	*
Rotation	0.00	**13.36**	5.81	**5.71**	1.66	*
	0.02	**20.27**	8.67	**11.26**	3.49	*
	0.04	**31.28**	18.3	**16.08**	10.12	*
	0.06	**78.58**	26.14	**34.27**	17.46	*

*difference statistically significant.

Rotation of the needle provided a larger tissue sample at all examined vacuum pressure levels regardless of the needle type. The differences were statistically significant at p < 0.01. Increasing the vacuum pressure from 0.00 to 0.06 MPa in 0.02 MPa steps raised significantly the mean sample weight regardless of the sampling methods and the needle type. The mean weight of the sample was bigger by a factor of 1.1–2.5 with each advancing step of increased vacuum pressure. The mean weight of tissue samples retrieved without aspiration was 7.55 mg, at 0.02 MPa it was 12.87 mg, at 0.04 MPa it reached 20.56 mg, and at the highest vacuum pressure of 0.06 MPa it increased to 39.34 mg. The samples obtained with different combination of biopsy parameters varied in mean weight by up to 20 times.

The risk of damaging the tissue from the specimen by high vacuum or mechanics of tissue sampling was evaluated with the Mair scale. The results are presented in Table 3.

**Table 3:** Diagnostic quality of biopsy tissue samples obtained with various combinations of biopsy technique after the Mair test. Each value is the mean of 10 histopathological evaluations.

		Blood/clot	Amount of cellular material	Degree of cellular degeneration	Degree of cellular trauma	Retention of appropriate architecture	Total
Needle	**Laitinen**	1.7	1.6	1.4	1.3	1.8	**7.8**
	**Nashold**	1.8	1.3	1.2	1.4	1.4	**7.0**
Sampling method	**Standard**	1.9	1.3	1.3	1.3	1.6	**7.4**
	**Rotation**	1.6	1.6	1.3	1.4	1.6	**7.4**
Vacuum pressure (MPa)	**0.00**	1.6	0.5	1.1	1.3	1.4	**5.9**
	**0.02**	1.9	1.4	1.0	1.3	1.5	**7.0**
	**0.04**	1.7	1.9	1.3	1.3	1.6	**7.7**
	**0.06**	1.9	2.0	1.8	1.6	2.0	**9.3**

The diagnostic quality of the slides obtained with different biopsy methods did not deteriorate with high vacuum or rotation of the needle during biopsy. All the samples were categorized as diagnostically superior. Only those obtained without vacuum fell under the category of “adequate for cytological diagnosis”. Larger samples obtained with higher vacuum pressure, needle rotation and the use of the Laitinen needle provided histological slides with higher diagnostic quality score. The samples obtained with the Laitinen needle and higher vacuum preserved better tissue cytoarchitecture.

The effectiveness of the biopsies in obtaining a sufficient amount of tissue for histopathological examination in relation to the needle type, sampling method or level of vacuum pressure is presented in Tables 4 and 5.

**Table 4:** The number of biopsy attempts necessary to obtain 50 tissue samples using various combinations of biopsy needles type, sampling method and levels of vacuum pressure.

Needle type	Sampling method	Vacuum pressure (MPa)	N^o^ of attempts delivering 50 samples	Effectiveness (%)	Mean effectiveness (%) regardless of pressure
Nashold	**Standard**	**0.00**	65	**76.9**	92.8
		**0.02**	52	**96.2**	
		**0.04**	51	**98.0**	
		**0.06**	50	**100.0**	
	**Rotation**	**0.00**	54	**92.6**	97.7
		**0.02**	50	**100.0**	
		**0.04**	51	**98.0**	
		**0.06**	50	**100.0**	
Laitinen	**Standard**	**0.00**	52	**96.2**	98.6
		**0.02**	51	**98.0**	
		**0.04**	50	**100.0**	
		**0.06**	50	**100.0**	
	**Rotation**	**0.00**	52	**96.2**	98.6
		**0.02**	51	**98.0**	
		**0.04**	50	**100.0**	
		**0.06**	50	**100.0**

**Table 5:** The cumulated effectiveness of biopsies related to the needle type, sampling method or level of vacuum pressure.

		N^o^ valid biopsies	N^o^ biopsy attempts	Effectiveness (%)	SD
Needle type	**Laitinen**	400	406	**98.50**	1.21
	**Nashold**	400	423	**94.25**	3.90
Sampling method	**Standard**	400	421	**94.25**	3.80
	**Rotation**	400	408	**98.00**	1.59
Vacuum pressure (MPa)	**0.00**	200	223	**88.50**	4.22
	**0.02**	200	204	**98.00**	0.89
	**0.04**	200	202	**99.00**	0.52
	**0.06**	200	200	**100.00**	0.00

Needle brain biopsy proved to be capable of delivering tissue specimens that were suitable for microscopic evaluation in 96.9% of attempts on average. The method has been proved effective in over 90% of the tested combinations of needle type, sampling method and vacuum level. The only exception is the Nashold needle standard method biopsy without vacuum pressure, which presented a lower level of effectiveness of 76.9%. An application of 0.06 MPa vacuum pressure delivered valid specimens in all attempts.

The number of expected valid biopsies was 400, delivered with each of the two needle types, 400 with each of the two sampling methods and 200 with each of the four vacuum pressures, regardless of the other measured factors. Biopsies with the Laitinen needle required fewer attempts than those with the Nashold needle to provide valid tissue samples. The difference of 4% points was small but statistically significant. Similarly, needle rotation was more effective than the standard method in 4% of the biopsies. The application of vacuum pressure significantly increased the effectiveness from 88.5% to 98%, with the lowest tested vacuum pressure of 0.02 MPa. The further elevation of vacuum pressure to 0.06 MPa increased the biopsy’s effectiveness only marginally, i.e. by a 1% point.

## Discussion

The role of brain biopsy in the clinical management of many brain diseases is widely emphasized upon in the literature. It is important in order to establish a definitive diagnosis and plan therapy^2–5,13,33–35^.

There are basically two aspects that limit the reliability of the brain needle biopsy results. The first one is related to the accuracy of targeting. The goal is to extract a sample from the core of the lesion and avoid tissue outside of the target area. This problem has already been extensively explored and greatly resolved by modern imaging and stereotactic armamentarium^5,11,16,17,19,25^. In the clinical reality, stereotactic brain needle biopsy is a predominant method to secure accurate diagnosis in the majority of cases: 72.8–95.6%^1,3–8^.

The second important aspect in obtaining an accurate diagnosis is related to the quality of tissue samples used in the preparation of histopathological slides. It has been established that the volume of biopsy specimen and its integrity are of paramount importance. A small size of tissue retrieved through needle biopsy as well as its fragmentation is a common limitation for all needle biopsies, resulting in interpretation challenges^10,33,36–38^. Larger tissue samples can ensure a better assessment of histologic architecture and facilitate molecular testing, which plays an increasingly important role in individualized medicine nowadays^39–41^. Jackson *et al*. indicated that a small quantity of tissue, commonly associated with stereotactic biopsies, were the most probable reason for observed diagnostic discrepancies^42^. This has been confirmed by Depreitere *et al*., who reported that the small size of biopsy samples was the primary reason for failure and problematic histological interpretation^11^. In a study by Torres *et al*., biopsies without useful data were substantially smaller in size than diagnostic biopsies (median 0.15 cm^3^ versus 0.4 cm^3^; P = 0.02)^43^.

Even though it is anticipated that the weight and integrity of biopsy specimens depend on the technique, this aspect has not been sufficiently investigated.

Through a review of the literature, it has been found that vacuum pressure has either not been used in brain needle biopsy, not been specified in the description of the biopsy method or has been defined as “slight suction” with a syringe. The authors of those studies admit that technical factors, such as the amount of negative pressure used to pull tissue samples into the biopsy window, were neither controlled for nor quantified^25–27,44,45^. Rossmeisl *et al*., in biopsy procedures using 16-gauge Nashold side-cutting needle, applied “slight negative pressure” with a syringe^44^. The appropriate amount of negative pressure required to obtain quality biopsy specimens was defined as a variable and influenced by the type of lesions sampled. The authors recommended that to harvest robust samples using side-cutting needles “slight negative pressure” should be applied^45^. Wani at al. applied suction through a 5-mL syringe at the base of the cannula. The actual pressure in this procedure was not measured^27^. Similarly, Flegel *et al*., in diagnosing encephalitis in dogs, applied negative pressure with a 0.5-mL syringe attached to the needle^25^.

In the present study, it was found that the biopsy sample’s size positively correlated with the increasing vacuum pressure. The increase of tissue sample was almost linearly related to the level of vacuum pressure. The level of vacuum pressure proved to be the main factor that influences the weight of the tissue specimen.

Similar effects were observed in one of the few published examinations on the influence of vacuum pressure on the volume of tissue obtained for *in-vitro* biopsies of cattle liver and swine testicles and kidneys. Those studies revealed an increase of sample mass following rising vacuum pressure, independent from the needle diameter and type of tissue^22,28^.

A well-designed study on the influence of vacuum pressure in needle aspiration and fine-needle *ex-vivo* liver biopsy was carried out by Haseler et at. They found that greater vacuum increased tissue samples yield but stressed that generating high vacuum pressure with hand-held syringes of various sizes increased the loss of control over the position of the needle tip^46^.

The papers published on brain needle biopsy employed the standard method of harvesting tissue protruding through the needle’s side window into the needle’s lumen. An inner cannula, acting as a guillotine, was used to cut the sample off. In this study, a novel method of obtaining tissue samples has been described and evaluated. It was named the rotation method. The samples taken with rotation were significantly larger at every vacuum level than those obtained employing the standard method. Increasing vacuum pressure in the rotation method consistently delivered samples of larger weight. This was more pronounced with the Laitinen needle than with the Nashold needle. According to our knowledge, this method has not been described before; therefore, there is no data available for comparison.

Brain needle biopsy, depending on the method of execution, may itself result in histopathological artefacts that influence diagnosis. This aspect has rarely been evaluated. Mechanical tissue damage during sampling has also been accused to be a potential factor affecting the accuracy of histopathological diagnosis^26^. According to Mahajan *et al*.^29^ and Mauryya *et al*.^30^, cellular degradation and trauma in the lymph node aspiration biopsies were more pronounced, whereas Mair *et al*.^31^ reported a better maintenance of cell architecture with aspiration. Higher cellularity of the preparations obtained with aspiration has been reported in a study by Chowhan *et al*.^21^. Kim *et al*. have investigated the possible untoward consequences of the methodology of brain biopsy on the histological quality of samples. They reported a semi-circular or band-like tissue compression in the periphery of the biopsy samples obtained with the side-cutting biopsy needle. This effect has been attributed to the mechanics of the side-cutting needle^26^.

The risk of structural damage to the fragile tissue resulting from high vacuum pressure and mechanical injury has been evaluated in this study using Mair’s methodology^31^. The samples mechanically separated, with and without the application of vacuum pressure of up to 0.06 MPa, from freshly isolated swine brains proved to be of a superior diagnostic quality. No histologically visible cell or tissue structure damage was found in the samples that were exposed to higher vacuum. On the contrary, due to the higher cellularity of the bigger samples obtained with suction and needle rotation, the diagnostic value of those slides was better. No difference in the diagnostic slide quality obtained with the Laitinen or Nashold needles at high vacuum pressures has been identified.

In the clinical setting, stereotactic brain biopsy fails to deliver tissue enabling histopathological evaluation (zero-biopsy) in around 10% of cases^5^. In the present experimental setting, increasing the vacuum pressure reduced the percentage of failed biopsies. It was found that biopsy effectiveness, meaning the retrieval of a tissue sample usable for evaluation, was reached in 97% cases on average. It was better when higher vacuum pressures were applied. Other technical aspects of the biopsy – needle type and sample separation method – influenced the effectiveness to a lesser extent. The Laitinen needle provided effective samples in 98.5% biopsies, 4.25% more than that by the Nashold needle. Rotation, notwithstanding other parameters, delivered around 4% more valid samples than the standard method.

The limitation of this study is related to the *ex-vivo* experimental setting. Mair’s score includes blood in the specimen as a disturbing factor. An experiment, even performed on a fresh *ex-vivo* tissue, does not adequately represent this factor. A planned study in living animals should give an answer on the risk of bleeding related to the biopsy technique.

This study applies to the biopsy of the brain without pathology. The results may not directly apply to the biopsy of pathological tissue of a different consistency, particularly that of higher cohesiveness.

The importance of this study’s results in performing brain needle biopsy is based on the conclusion that substantially larger tissue samples are obtained with the needle rotation method and vacuum application. Tissue retrieval using vacuum pressure of up to 0.06 MPa and needle rotation does not deteriorate the pathological diagnostic quality of the samples. To the best of our knowledge, this is the first study on the effects of precisely defined vacuum pressure and needle rotation on the efficacy of brain needle biopsy.

## Conclusions

Brain tissue samples obtained through needle biopsy are better for histopathological evaluation when obtained with higher vacuum pressure, a novel needle rotation method and with Laitinen needle.

Higher suction pressure and sampling methods did not cause tissue damage.
